# Prognostic Utility of Cytotoxic T-Lymphocyte Antigen 4 (CTLA-4) Expression in Head and Neck Squamous Cell Carcinoma: A Retrospective Study

**DOI:** 10.7759/cureus.88775

**Published:** 2025-07-25

**Authors:** Aishwarya Mohan, Barathi Gunabooshanam, Sandhya Sundaram, Banu Keerthana

**Affiliations:** 1 Clinical Immunology, Sri Ramachandra Institute of Higher Education and Research, Chennai, IND; 2 Pathology, Sri Ramachandra Institute of Higher Education and Research, Chennai, IND

**Keywords:** ctla-4, head and neck squamous cell carcinoma, immune checkpoints, immunohistochemistry, immunotherapy, prognosis

## Abstract

Purpose

Head and neck squamous cell carcinomas (HNSCCs) represent a significant oncological challenge, with diverse anatomical presentations and variable clinical outcomes. Immune checkpoints, particularly cytotoxic T-lymphocyte antigen-4 (CTLA-4), play a pivotal role in regulating the immune response and may influence tumor progression and treatment response.

Methods

In this retrospective study, we aimed to assess the expression of CTLA-4 in paraffin-embedded HNSCC tissue samples and investigate its prognostic utility. Immunohistochemical analysis was planned to be performed on 50 patient samples to assess a significant association between CTLA-4 expression and tumor characteristics.

Results

Males exhibited a higher prevalence of CTLA-4 expression compared to females. The intensity of CTLA-4 staining correlated with the proportion of tumor distribution, with statistically significant differences observed between different intensity scores. Additionally, a significant relationship between CTLA-4 expression and tumor grade, particularly in moderately differentiated tumors (G2), was identified (p = 0.000). However, negative staining and membrane-cytoplasm staining lacked statistical significance.

Conclusion

Our findings suggest that CTLA-4 expression in HNSCC is associated with tumor progression to high-grade neoplasia and may serve as a marker of tumor biological behavior. Further research correlating CTLA-4 expression with other prognostic markers, such as p16, and its implications for immunotherapy in HNSCC are warranted.

## Introduction

Head and neck squamous cell carcinoma (HNSCC) constitutes a heterogeneous group of epithelial neoplasms arising in the upper aerodigestive tract [1]. Concurrently, squamous cell carcinoma (SCC) ranks as the second most common type of skin cancer, typically emerging on sun-exposed regions, including the head, neck, and extremities, often due to UV radiation exposure [2]. The prevalence of HNSCC exhibits regional variation and is frequently associated with factors such as excessive alcohol consumption, cigarette smoking, or both.

Early-stage localized disease accounts for approximately 40% of HNSCC cases, necessitating multimodality treatment approaches [3]. Diagnosis of HNSCC typically entails a neck mass or primary tumor biopsy, with the biopsy technique contingent on lesion location. Mucosal epithelial cells lining the oral cavity, tongue, larynx, pharynx, and sinonasal tract serve as the origin of HNSCC, progressing from epithelial cell hyperplasia to invasive carcinoma [4]. Notably, a history of pre-malignant lesions is uncommon among HNSCC patients.

Cytotoxic T-lymphocyte antigen-4 (CTLA-4), a cell surface protein predominantly expressed on T cells, including regulatory T cells (Tregs) and effector T cells (Teff), plays a pivotal role in immune regulation. CTLA-4 interacts with antigen-presenting cells (APCs) expressing CD80 (B7-1) and CD86 (B7-2), thereby inhibiting T cell activation by blocking T cell receptor (TCR) signaling [5]. Activation of this immunological checkpoint modulates Treg activity and downregulates CD4+ T helper function.

Monoclonal antibodies that block CTLA-4 induce tumor regression. Therapeutic immunity against cancer is enhanced in HNSCC patients with higher intra-tumoral T-regulator inhibition of CTLA-4 cells. The enhanced immunosuppressive activity of Tregs, the first negative regulators of T cell activation in antitumor immunity, is ascribed to transforming growth factor beta 1 (TGF-β1) mechanisms. CTLA-4 is a negative regulator of T cell activation in the context of antitumor immunity; in preclinical models, monoclonal antibody-mediated inhibition of CTLA-4 leads to tumor regression and long-lasting antitumor immunity [6]. CTLA-4 expression is higher in HNSCC tumor samples compared to normal tissue, although it is not associated with pathological tumor grade or lymph node metastases [7]. This retrospective study aimed to assess the CTLA4 expression in tissue samples from HNSCC that were paraffin-embedded and to assess the expression with various parameters, such as the proportion of the distribution of tumors and tumor grade.

## Materials and methods

Study design and period

This was a retrospective study conducted in a tertiary care hospital in the southern part of India, between December 2021 and January 2023.

Study population

The investigation was conducted on a sample of 50 cases with histopathologically confirmed HNSCC, using formalin-fixed, paraffin-embedded (FFPE) tissue blocks.

Inclusion criteria included histologically confirmed, non-human papillomavirus (HPV)-related squamous cell carcinoma with available FFPE tissue and complete clinical data. Exclusion criteria comprised cases with HPV-positive status, non-squamous histology benign lesions.

Ethical approval and informed consent

Approval from the institutional ethics committee was obtained (REF: CSP/23/APR/126/306) before the commencement of the study.

Operational definition and data collection tools

The paraffin-embedded tissue blocks were used for immunohistochemistry (IHC). A monoclonal anti-CTLA-4 antibody (clone UMAB249, OriGene Technologies Inc., Rockville, MD; dilution 1:100) was used, with antigen retrieval performed in Tris buffer (pH 6.0) by a pressure cooker for 20 minutes. Immunohistochemistry (IHC) was carried out using a polymer-based detection system (horseradish peroxidase (HRP)-conjugated secondary antibody with 3,3′-diaminobenzidine (DAB) chromogen), and counterstaining this method was used for detection after the slides had been incubated with the primary antibody for 60 minutes at room temperature before detection and counterstaining.

IHC with CTLA-4 antibody was done after proper standardization. As a control for the test, a tonsillar tissue was employed. The intensity of staining is based on the values of (1+) negative, (2+) weak, (3+) moderately positive, and (4+) strongly positive. The Q score was calculated by multiplying the intensity of staining by the percentage of positive tumor cells (Q score = % of positive cells × intensity score).

Statistical analysis 

Statistical Product and Service Solutions (SPSS, version 23.0; IBM SPSS Statistics for Windows, Armonk, NY) software was used to examine the data. Frequency (N) and percentage (%) were employed in the assessment of quantitative parameters. Chi-square analysis was used to assess frequency distribution.

## Results

**Figure 1:**
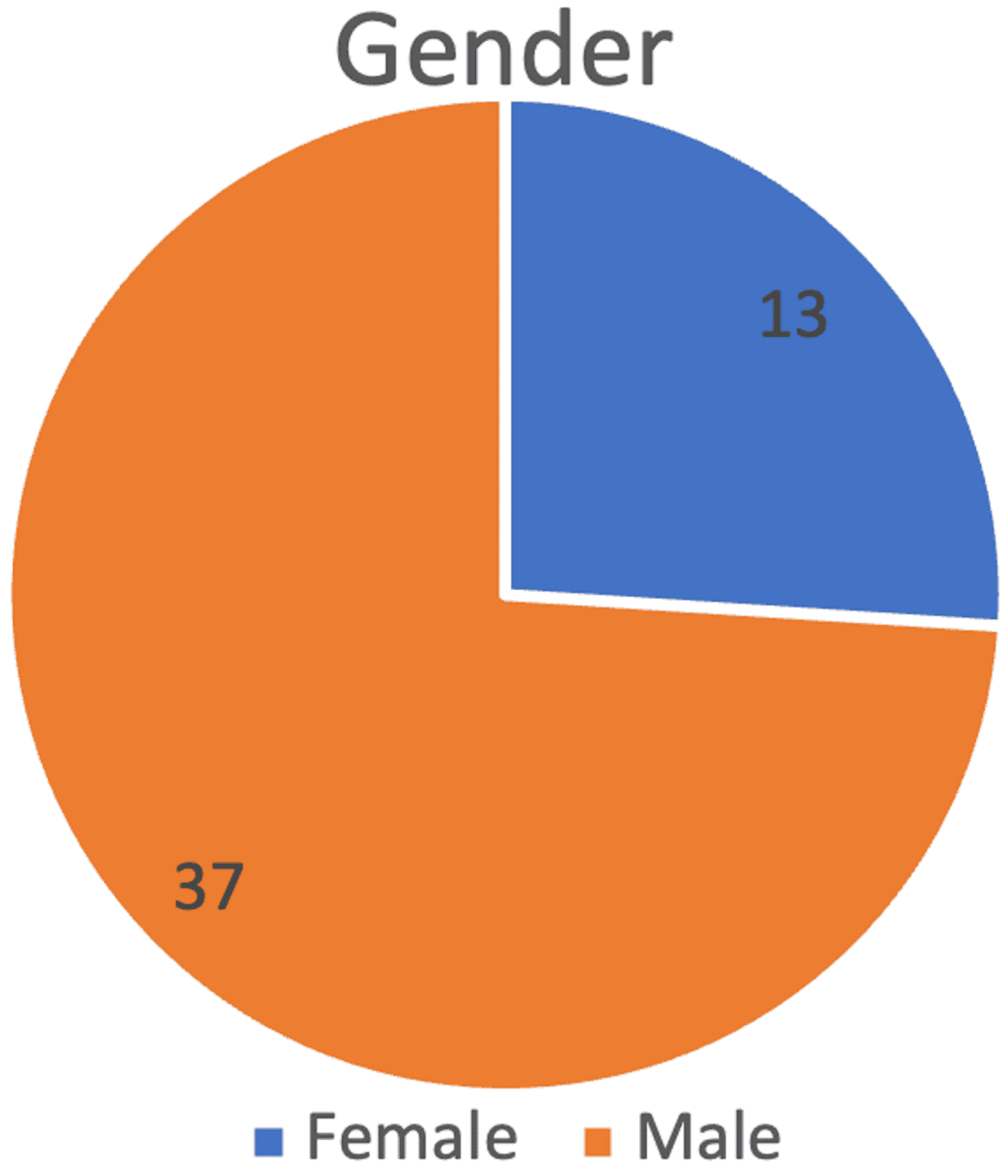
Gender distribution of the study sample showing male predominance Data have been represented as N.

During our study period from December 2021 to January 2023, a total of 50 cases of HNSCC were analyzed. To ensure objectivity in immunohistochemical scoring, two independent pathologists scored the CTLA-4-stained slides. Both were blinded to clinical outcomes. Among these, 74% (n = 37) were male and 26% (n = 13) were female, resulting in a male-to-female ratio of 2.85:1, indicating a significant male predominance (Figure 1). Out of 50 HNSCC cases, the site distribution was as follows: oral cavity (N = 33), hypopharynx (N = 2), nasopharynx (N = 3), larynx (N = 7), and oropharynx (N = 5). Most cases were in the oral cavity, followed by the larynx and oropharynx.

**Figure 2:**
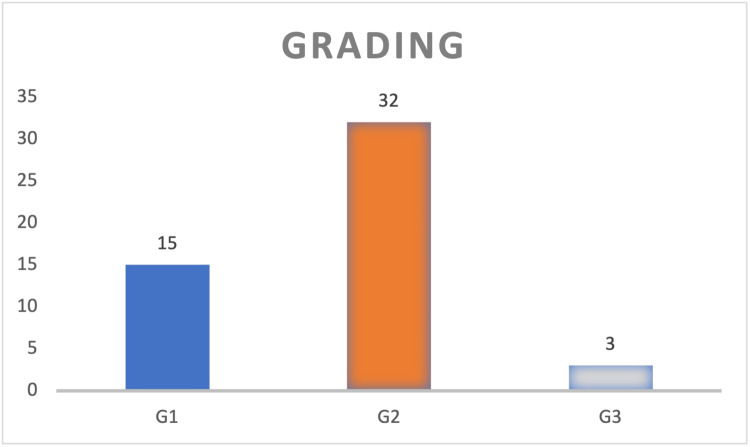
Distribution of HNSCC cases according to histological grades HNSCC: head and neck squamous cell carcinoma G1 (well differentiated): 15 cases, representing 30% (n=15) of the overall sample. G2 (moderately differentiated): 64% (N = 32) of the sample fell into this group. G3 (poorly differentiated): Only three instances (6%, N = 3) of the sample fall under this category. The data have been represented as N.

The "Grading" bar graph (Figure 2) depicts the distribution of HNSCC patients according to their histological grades (G1, G2, G3). The x-axis categorizes the grades, while the y-axis represents the number of cases. The findings indicate that the majority of HNSCC patients in this study were moderately differentiated (G2), with a smaller proportion being well differentiated (G1) or poorly differentiated (G3). This distribution reveals a significant tendency toward moderate differentiation in tumor grade in the study group.

The research sample had a male-to-female ratio of approximately 2.85:1. Most tumors were moderately differentiated (G2), accounting for 64% (N = 32). Well-differentiated tumors (G1) accounted for 30% (N = 15), whereas poorly differentiated tumors (G3) were the least prevalent, with just 6% (N = 3).

**Figure 3:**
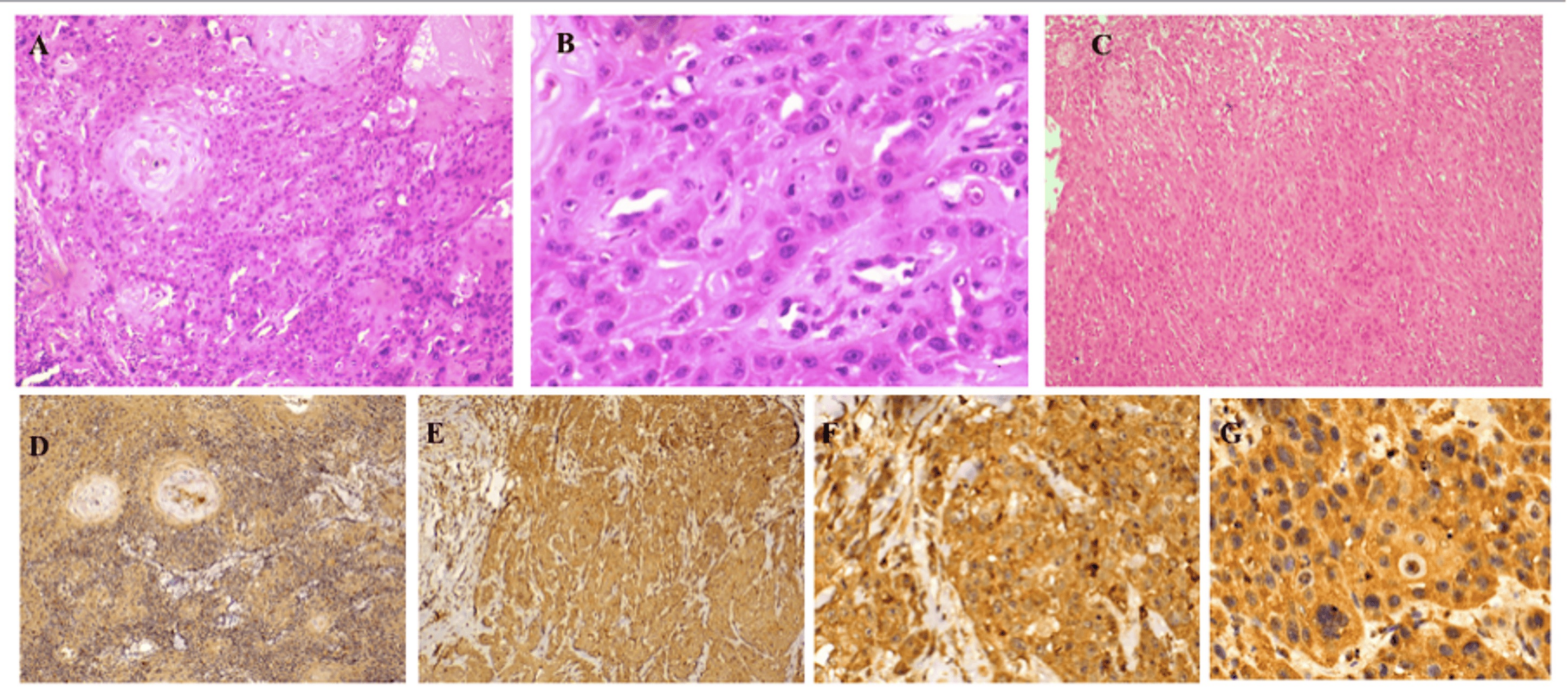
A. Well differentiated squamous cell carcinoma, H&E (200x). B. Moderately differentiated squamous cell carcinoma, H&E (200x). C. Poorly differentiated squamous cell carcinoma, H&E (100x). D. Well differentiated squamous cell carcinoma, Immunohistochemistry CTLA4 staining 1+intensity (100x). E. Moderately differentiated squamous cell carcinoma, CTLA4 staining 2+ intensity (100x). F. Moderately differentiated squamous cell carcinoma, CTLA4 staining 3+ intensity (200x). G. Moderately differentiated squamous cell carcinoma, CTLA4 staining 4+ intensity CTLA-4: cytotoxic T-lymphocyte antigen-4; HNSCC: head and neck squamous cell carcinomas

By IHC analysis of CTLA-4 expression, positive staining was defined as distinct membranous and/or cytoplasmic brown coloration observed in tumor cells, regardless of intensity. Negative staining was considered when no specific immunoreactivity was observed in tumor cells, even in the presence of background staining in stromal or inflammatory cells. Only tumor cells were considered for positivity evaluation, in which 23 samples (46%) were negative for staining. Of the 27 positive samples, 26% (N = 13) had cytoplasmic staining; 22% (N = 11) had both cytoplasmic and membranous staining, and 6% (N = 3) showed membranous staining only. Staining intensity varied, with 23 samples showing 1+ intensity (46%), 19 samples showing 2+ intensity (38%), seven samples showing 3+ intensity (14%), and one sample showing 4+ intensity (2%) (Figure 3). Tumor cell percentage stained: variations were seen among patients. Notably, 23 samples (46%) showed 100% staining. Lower percentages included six (12%) samples with 10% staining, with the remaining samples showing percentages ranging from 2% to 12%. Quantitative scoring (Q scoring) revealed that 23 samples (46%) were negative. The Q values for positive cases varied from 0.2 to 2.4, with 8 samples (16%) scoring 1.6.

Lymphocyte infiltration

Scores varied, with 20% (N = 10) scoring 0.1, and 18% (N = 9) scoring 0.3 and 0.4, and lower rates for others. Only one sample (2%) tested negative for lymphocyte infiltration. The research had a somewhat greater number of patients under 60 years old, with 58% (N = 29), and 42% (N = 21) over 60 years old (Table 1).

**Table 1:** Frequency distribution of variables HNSCC: head and neck squamous cell carcinoma The table shows the frequency distribution of major demographic, clinical, and pathological factors in a sample of 50 patients diagnosed with HNSCC. The data are presented as frequency (N) and percentage (%).

Variables		Frequency	Percentage
Gender	Female	13	26
	Male	37	74
Grading	G1	15	30
	G2	32	64
	G3	3	6
Staining	Cytoplasm	13	26
	Cytoplasm and membrane	11	22
	Membrane	3	6
	Negative	23	46
Intensity of score	1+	23	46
	2+	19	38
	3+	7	14
	4+	1	2
% tumor cells	10%	6	12
	20%	1	2
	30%	6	12
	40%	5	10
	60%	2	4
	70%	1	2
	80%	6	12
	100%	23	46
	Q scoring	0.2	6	12
0.6		5	10
0.8		6	12
1.6		8	16
2.4		2	4
Negative		23	46
Lymphocytes	0.1	10	20
	0.2	3	6
	0.3	9	18
	0.4	9	18
	0.5	2	4
	0.6	3	6
	0.8	2	4
	Negative	1	2
Age	<60	29	58
	>60	21	42

Correlation between the intensity of staining and the percentage of tumor cells

A statistically significant association was discovered, with a p < 0.001, suggesting that staining intensity is strongly related to the percentage of tumor cells (Table 2). All 23 samples with a 1+ intensity had 50% to 100% of tumor cells stained, with none falling between 10% and 50%. Most patients with 2+ intensity (N = 14) had 10% to 50% tumor cell staining, while only 5 had 50% to 100%. Cases with 3+ intensity were more evenly distributed: 42.9% (N = 3) in the 10% to 50% group and 57.1% (N = 4) in the 50% to 100% group. The only patient with 4+ intensity had 10% to 50% tumor cells stained. These findings indicate that lower intensity values (1+) are associated with a higher percentage of tumor cells stained, while higher intensity scores (2+, 3+, 4+) show a more varied distribution.

**Table 2:** Highlights the relationship between the intensity of IHC staining and the proportion of tumor cells stained IHC: immunohistochemistry Table shows the intensity score of IHC staining and the proportion of tumor cells stained. Chi-square test shows p < 0.001.

Intensity of score	% Tumor cells		P value	Chi^2 ^- Numerical values
	10%-50%	50%-100%		
1+	0 (0%)	23 (100%)	<0.0001	
2+	14 (73.8%)	5 (26.3%)		26.57
3+	3 (42.9%)	4 (57.2%)		
4+	1 (100%)	0 (0%)		

As the association between age and various clinicopathological variables like tumor grading, staining pattern, intensity score, percentage of tumor cells, Q scoring, and lymphocyte levels, no statistically significant associations were observed across variables, as indicated by the P values (>0.05) (Table 3).

**Table 3:** Association between age and variables This table represents the variables, including grading, staining, intensity of score, % tumor cells, Q scoring, and lymphocytes between the age groups less than and more than 60 years. A chi-square t-test was done with P value <0.05 was considered significant.

Variables		Age		P value	Statistical test	Chi^2^-Numerical values
		<60	>60			
Grading	G1	10 (34.4%)	5 (23.8%)			
	G2	18 (62.0%)	14 (66.7%)			
	G3	1 (3.4%)	2 (9.6%)			
Staining	Cytoplasm	7 (24.1%)	4 (19.0%)	0.66	Chi-square test	2.4
	Cytoplasm and membrane	2 (6.9%)	0 (0%)			
	Membrane	5 (17.2%)	6 (28.6%)			
	Negative	2 (6.9%)	1 (4.8%)			
Intensity of score	1+	13 (44.8%)	10 (47.6%)	0.685	Chi-square test	1.49
	2+	10 (34.5%)	9 (42.9%)			
	3+	5 (17.2%)	2 (9.5%)			
	4+	1 (3.4%)	0 (0%)			
% tumor cells	10%	3 (10.3%)	3 (14.3%)	0.448	Chi-square test	6.82
	20%	0 (0%)	1 (4.8%)			
	30%	4 (13.8%)	2 (9.5%)			
	40%	2 (6.9%)	3 (14.3%)			
	60%	2 (6.9%)	0 (0%)			
	70%	0 (0%)	1 (4.8%)			
	80%	5 (17.2%)	1 (4.8%)			
	100%	13 (44.8%)	10 (47.6%)			
Q scoring	0.2	3 (10.3%)	3 (14.3%)	0.562	Chi-square test	3.91
	0.6	2 (6.9%)	3 (14.3%)			
	0.8	3 (10.3%)	3 (14.3%)			
	1.6	7 (24.1%)	1 (4.8%)			
	2.4	1 (3.4%)	1 (4.8%)			
	Negative	13 (44.8%)	10 (47.6%)			
Lymphocytes	0.1	4 (13.8%)	6 (28.6%)	0.425	Chi-square test	8.11
	0.2	2 (6.9%)	1 (4.8%)			
	0.3	6 (20.7%)	3 (14.3%)			
	0.4	5 (17.2%)	4 (19.0%)			
	0.5	2 (6.9%)	0 (0%)			
	0.6	3 (10.3%)	0 (0%)			
	0.8	2 (6.9%)	0 (0%)			
	Negative	5 (17.2%)	7 (33.3%)			

## Discussion

HNSCCs are characterized by immune suppression mediated through key immune checkpoint pathways, particularly CTLA-4. CTLA-4 is a crucial regulator expressed on T cells that binds with CD80 and CD86 on APCs to modulate immune responses. This study explored the prognostic significance of CTLA-4 expression in paraffin-embedded HNSCC tissue samples, contributing to the growing body of research that positions CTLA-4 as a pivotal target for immunotherapy [8,9].

In the present study, we found a significant correlation between CTLA-4 intensity scores and the percentage of tumor distribution within paraffin-embedded HNSCC tissue samples. Tumor regions exhibiting 10-50% distribution predominantly showed an intensity score of 2+ (73.8%), while those with 50-100% distribution were associated with an intensity score of 1+ (100%). Similar observations have been reported, where CTLA-4 expression was found to be heterogeneous within and across tumors. These findings underline the critical involvement of CTLA-4 in modulating the tumor microenvironment [10].

Interestingly, another significant finding was that grade 2 tumors (moderately differentiated SCC) showed higher expression of CTLA-4. This is in concordance with Balci et al. (2021), who observed that 31% of SCCs had strong CTLA-4 expression, with prevalence in grades I, II, and III being 43%, 31%, and 27%, respectively. However, their results indicated a pattern for lower grades to have more CTLA-4, and there was no statistically significant (p > 0.05) difference. This implies that moderately differentiated tumors may exhibit high levels of CTLA-4 communication, which could reflect tumor immune evasion pathways that are active at these levels [11]. An age comparison with CTLA 4 expression in the tumor was done in our study, which did not show any significant correlation. This finding was in concordance with the study of Johnson et al. [4].

Negative staining patterns and cytoplasmic membrane staining were not statistically significant in our group (p > 0.05). This aligns with findings by Lan et al., who reported that in breast cancer, neither interstitial nor tumor CTLA-4 expression was linked to any clinical parameters, including tumor grade, supporting the lack of significant correlation between CTLA-4 staining patterns and clinicopathological features [12]. No significant association between negative staining or membrane-cytoplasm localization of CTLA-4 and tumor grade, as reflected by non-significant p-values. Though gender comparison with CTLA4 expression was not performed in our study, Yusnita et al. (2025) a study on diffuse large B-cell lymphoma (DLBCL) patients, found higher CTLA-4 expression in females compared to males [13].

Limitation

Being a retrospective, single-center study, the relatively small sample size of 50 cases may limit the statistical power and robustness of the findings. The absence of correlation with clinical outcomes such as treatment response, recurrence, or survival further limits the prognostic applicability of the findings. Comprehensive profiling of the tumor microenvironment was not performed.

## Conclusions

Our findings indicate that CTLA-4 expression in HNSCC plays an important role in tumor progression toward higher-grade neoplasia and may be linked to tumor biological behavior. CTLA-4, primarily present on T cells, interacts with CD80 and CD86 on APC surfaces. When compared to other grades, it indicates a high-grade range (G2). CTLA-4 expression is quite frequent in HNSCC. HPV-associated HNSCC typically expresses P16, which may be evaluated with IHC and has strong prognostic value in immunotherapy. Further research should include an assessment of CTLA4 and the p16 association.
